# Assessment of Migration of Human MSCs through Fibrin Hydrogels as a Tool for Formulation Optimisation

**DOI:** 10.3390/ma11091781

**Published:** 2018-09-19

**Authors:** Nasseem Salam, Sotiria Toumpaniari, Piergiorgio Gentile, Ana Marina Ferreira, Kenneth Dalgarno, Simon Partridge

**Affiliations:** 1 School of Medicine Medical Sciences and Nutrition, University of Aberdeen, King’s College, Aberdeen AB24 3FX, UK; r02ns17@abdn.ac.uk; 2 Department of Materials Science and Metallurgy, University of Cambridge, 27 Charles Babbage Road, Cambridge CB3 0FS, UK; st684@cam.ac.uk; 3 School of Engineering, Newcastle University, Newcastle upon Tyne NE1 7RU, UK; Piergiorgio.Gentile@newcastle.ac.uk (P.G.); Ana.Ferreira-Duarte@newcastle.ac.uk (A.M.F.); 4 Materials and Engineering Research Institute, Sheffield Hallam University, City Campus, Howard Street, Sheffield S1 1WB, UK; S.Partridge@shu.ac.uk

**Keywords:** fibrin hydrogels, 3D cell migration, stem cells, cell delivery, regenerative medicine

## Abstract

Control of cell migration is fundamental to the performance of materials for cell delivery, as for cells to provide any therapeutic effect, they must migrate out from the delivery material. Here the influence of fibrinogen concentration on the migration of encapsulated human mesenchymal stem cells (hMSCs) from a cell spheroid through fibrin hydrogels is tracked over time. Fibrin was chosen as a model material as it is routinely employed as a haemostatic agent and more recently has been applied as a localised delivery vehicle for potential therapeutic cell populations. The hydrogels consisted of 5 U/mL thrombin and between 5 and 50 mg/mL fibrinogen. Microstructural and viscoelastic properties of different compositions were evaluated using SEM and rheometry. Increasing the fibrinogen concentration resulted in a visibly denser matrix with smaller pores and higher stiffness. hMSCs dispersed within the fibrin gels maintained cell viability post-encapsulation, however, the migration of cells from an encapsulated spheroid revealed that denser fibrin matrices inhibit cell migration. This study provides the first quantitative study on the influence of fibrinogen concentration on 3D hMSC migration within fibrin gels, which can be used to guide material selection for scaffold design in tissue engineering and for the clinical application of fibrin sealants.

## 1. Introduction

Hydrogels, highly hydrated cross-linked polymers, are increasingly sought as tissue engineering scaffolds due to their hydrophilicity, permissive nutrient/waste exchange, and predisposition for biocompatibility [1,2].

Fibrin, a protein biopolymer that simulates the final stage of the coagulation cascade, is routinely employed as a haemostatic agent [3]. The polymer network is generated following addition of thrombin, a serine protease, to the 45 nm monomer fibrinogen [4]. Fibrin is used in augmenting wound healing, and is increasingly used to promote angiogenesis [5], to release drugs and growth factors [6], and as a mode for cell delivery [3,7], with commercial fibrin sealants used to encapsulate cells for delivery [8,9,10]. Encapsulation within a gel for delivery into the body is an attractive option as it allows cells to be positioned within the body, and temporarily retained in position, but in order for the cells to offer a therapeutic effect they need to then migrate out of the gel and interact with native tissue. Adipose-derived human mesenchymal stem cells (hMSCs) have been delivered in fibrin to augment microfracture procedures in cartilage regeneration [11], with histological evaluation demonstrating superiority over the microfracture-only procedure. hMSCs are commonly identified as cells of particular interest for tissue engineering [12,13].

Fibrin’s non-toxic biodegradability, abundance, low cost, potential for autologous origin, and adjustable mechanical and physical properties gives the material some advantages over other hydrogels [3]. The diverse adoption of fibrin hydrogels testifies to their versatility with applications in musculoskeletal [14,15,16], skin [17], cardiovascular [18,19], neural [20], and ocular [21,22] tissue engineering.

Conventional migration assays are constrained by their two-dimensionality [23]. Often composed of cells cultured as a monolayer upon the investigated substrate, these assays provide a simplified overview of in vivo cell migration with ensuing disparities when translating research into the clinic [24]. Migration studies on 2D substrates and/or tissue culture plastics introduces a number of artificial elements including high stiffness, cell contacts restricted to *x*- and *y*-planes, abnormal polarisation, and the absence of biophysical impediments found in native tissue [25]. Indeed, a growing body of work attests to distinct mechanical, physical, and biochemical processes that occur in a 3D microenvironment [26,27,28]. Research into simulating in vivo cell migration has highlighted the diverse modes of migration for different cell types according to their anatomical location and physiological role [24]. Generally, cells migrate in a phased process involving: (1) polarisation inducing the formation of a leading edge comprising cytoskeletal projections, (2) attachment onto the matrix and maturation of cell-matrix interactions, and (3) detachment at the posterior to move forward [24,29]. Moreover, migration through matrices featuring physical impediments requires an adjustment in cell shape, proteolytic degradation, and/or subsequent cell-mediated remodelling of the microenvironment [25].

With respect to fibrin, modification of precursor concentrations alters the hydrogel architecture which consequently affects the rate of migration; encapsulated cells migrate by physically advancing through size-permitting pores or secreting enzymes such as matrix metalloproteinases (MMPs) to remodel the matrix [30,31,32]. As a protein, fibrin undergoes degradation, principally by way of plasmin and MMP activity [25,33]. In general, gelation kinetics determines gel structure which can be configured by adjusting concentrations of fibrinogen [34], thrombin [35], and Ca^2+^ [36], Factor XIIIa [37], the local ionic strength, pH, and temperature [38,39]. Differing views exist regarding the extent to which the aforementioned factors induces the greatest effect on gel structure and consequently, cell behaviour including viability, proliferation, and migration. Nevertheless, the predominant role of fibrinogen concentration has been well-documented [7,34,40].

Although there has been considerable investigation into fibrin hydrogel-encapsulated cells, little information on 3D cell migration is available. Earlier research into migration and fibrin aimed to further understanding of the wound healing process [41,42]. In these investigations, cultured monolayers on fibrin substrates were incised as characteristic of a scratch assay to assess migration. Of relevance, Schleef et al. highlighted the influence of fibrinogen concentration on endothelial cell migration [42], noting that 2D migration was reduced as fibrinogen concentration increased. Other studies have encapsulated cells within 3D fibrin gels and examined cell outgrowth into the surrounding tissue culture plastic [34,43]. While cells were surrounded in a fibrin matrix, these studies nevertheless investigated migration from a 3D gel onto a 2D well plate. Recently, Hakkinen et al. assessed the migration of encapsulated neurites within four 3D ECMs including fibrin gels [44], and Nandi and Brown [45] assessed the effect of pro- and anti-migratory factors on fibroblast migration from encapsulated spheroids. However, in both cases gel formulations were kept constant and thus, no examination on the effect of precursor concentrations was carried out. Interestingly, increasing fibrinogen concentration reduced neurite extension from dorsal root ganglions embedded in fibrin gels [46]. The researchers underlined the impact of fibrin’s physical properties including gel stiffness and porosity—both concomitant factors in cell migration—on neurite extension.

In 2D studies of MSC behaviour using different concentrations of fibrinogen and thrombin, both Ho et al. [40] and Hale et al. [47] found that the MSC proliferation rate was affected by fibrinogen and thrombin concentrations, finding that cells proliferate faster with dilute fibrinogen solutions. Similarly, Catelas et al. analysed MSC behaviour (morphology, proliferation and differentiation) at eight different fibrin concentrations [48]. However, in both studies migration rates were not investigated. The aim of the work reported here was to systematically assess the ability of hMSCs to migrate within 3D fibrin hydrogels in order to inform clinical practice, specifically on the use of fibrin sealants for cell delivery, and in addition to illustrate the value of 3D migration models as a gel formulation optimisation tool for tissue engineers.

## 2. Materials and Methods

### 2.1. Fibrin Gel Preparation and Synthesis

Fibrin gels were prepared from plasma-derived bovine fibrinogen (341573, Merck Millipore, Watford, UK) and bovine thrombin (605157, Merck Millipore, Watford, UK). Broadly, fibrinogen was reconstituted in Hank’s buffer salt solution while thrombin was reconstituted in a diluent solution comprising hMSC cell culture media (with composition as described in Section 2.4) modified to contain 20 mM calcium chloride. All components were pre-warmed to 37 °C and gently mixed to ensure complete solubilisation.

**Figure 1:**
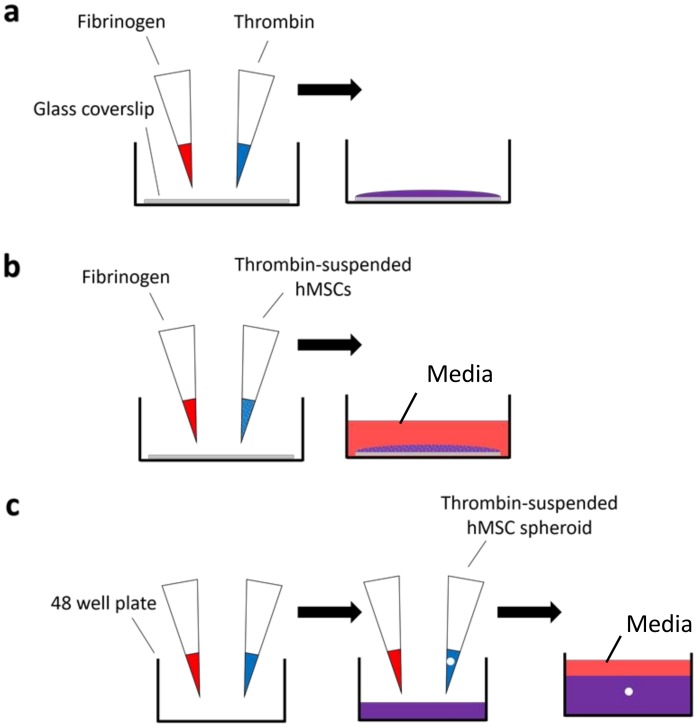
Schematic overview of fibrin formation for (**a**) material characterisation experiments, (**b**) encapsulated hMSCs in fibrin gels, and (**c**) encapsulated hMSC spheroids in fibrin gels for migration experiments. Fibrin gels were formed by mixing fibrinogen (red) and thrombin (blue) in equal volumes on parafilm before deposition onto glass coverslips (**a**,**b**) or tissue culture plastic (**c**). Once formed, gels were allowed to gelate at 37 °C/5% CO_2_ for 1 h before addition of media.

Fibrin gels with final concentrations of 5 mg/mL to 50 mg/mL fibrinogen and 5 U/mL thrombin were used, based on concentrations used in the literature and to give a good range of properties. Fibrin formation was initiated by mixing fibrinogen at the different concentrations and thrombin in equal volumes before allowing for gelation at 37 °C/5% CO_2_ in a humid atmosphere for 1 h. Depending on the characterisation, materials were prepared using one of three different procedures (outlined schematically in Figure 1):

(a) A total of 200 μL of formed fibrin gel material was deposited on borosilicate glass coverslips for the physical-chemical characterisations.
(b) For the different cellular studies, cells were added within thrombin solution and fibrin gels containing 25,000 cells/gel were formed on 13 mm diameter circular borosilicate glass coverslips (631-0159, VWR, Lutterworth, UK).
(c) For cell migration experiments, cellular spheroids (50,000 cell) were encapsulated within fibrin gels in multi-wells.

Glass coverslips were plasma treated by using a PDC-32G plasma cleaner (Harrick Plasma, Ithaca, NY, USA) for an improved surface wettability and subsequently sterilised under UV light at 254 nm for 30 min. Each experiment was performed in triplicate.

### 2.2. ScanningElectron Microscopy

Formed fibrin gels were washed in distilled water thrice for 15 min each to remove excess salt and fixed in 2.5% glutaraldehyde (v/v) in phosphate buffer saline (PBS) solution for 2 h at 4 °C. Gels were dehydrated in a graded series of 50%, 75%, and two 100% (v/v) ethanol/distilled water solutions for 15 min each. Dehydrated gels were placed in a Baltec CPD 030 critical point dryer (Leica Microsystems, Milton Keynes, Watford, UK) to replace ethanol with liquid CO_2_ before removal. Once dried, gels were mounted on aluminium stubs, sputter-coated with approximately 10 nm gold using a Polaron E5000 sputter coater (Quorum Technologies, Laughton, UK), and observed under a Tescan VEGA LMU SEM microscope (Tescan, Cambridge, UK) with an accelerating voltage of 8.0 kV and working distance of 8–10.0 mm. Images were then analysed using ImageJ. Images at 25 k magnification were divided into four quadrants, and within each quadrant three representative fibre or pore diameters were measured. Two images for each fibrinogen concentration were assessed giving 24 measurements for statistical analysis.

### 2.3. Rheological Characterisation

The viscoelastic properties of the fibrin gels on glass coverslips were determined using a Malvern Kinexus pro + rotational rheometer (Malvern Instruments Ltd., Malvern, UK) configured with a parallel plate geometry (20 mm upper platen with a measuring gap of approximately 1.5 mm, preloaded to 0.1 N). Temperature was maintained with a high temperature cartridge at 37 °C while evaporation was controlled with a solvent trap filled with distilled water. Oscillatory experiments were performed with strain sweeps (0.01–100% strain, 1 Hz) to assess the rheological properties of the gels.

### 2.4. Cell Culture

Immortalised TERT-hMSCs. developed at the University of York (Y201 line, as described in [49]) were used as they provide a consistent source of hMSCs for experimentation. The cells were routinely cultured in Dulbecco’s modified Eagle’s medium with 1 g/L glucose (BE12-707F, Lonza, Slough, UK) supplemented with 10% (v/v) foetal bovine serum (Gibco A3160402, ThermoFisher Scientific, Loughborough, UK), 1% (v/v) penicillin and streptomycin (P4333, Sigma Aldrich, Gillingham, UK), and 5 mM L-glutamine (G7513, Sigma, Gillingham, UK). Cells were maintained from passage 76 in routine cell culture at 37 °C/5% CO_2_, and were passaged when judged to be 70–80% confluent.

### 2.5. Live/Dead Cell Staining

The morphology and viability of hMSCs encapsulated in fibrin gels (25,000 cells/gel) was assessed using live/dead cell staining to visualize the viable (green) and dead (red) cells. Cells grown on glass coverslips (25,000 cells/coverslip) served as a control. Six hours after gelation, cell-laden fibrin gels were washed in PBS for 30 min before incubation with 100 μL of live/dead stain containing 2 μM calcein-AM and 4 μM ethidium homodimer-1 (Thermo Fisher Scientific, Loughborough, UK) for a further 30 min. Gels were incubated at 37 °C/5% CO_2_ during washing and incubation. Following incubation, cell-laden fibrin gels were imaged under a Leica M165 FC stereo microscope (Leica Microsystems, Milton Keynes, UK) at 100× magnification; calcein-AM was examined at BP 450–490 nm excitation and LP 515 nm emission, ethidium homodimer-1 was examined at BP 515–560 nm excitation and LP 590 nm emission. Representative images were acquired with LAS Core software (Leica Microsystems, Milton Keynes, UK) at the center and edges of each gel (images taken at day 0 with 3 samples per concentration).

### 2.6. PrestoBlue^®^ Cell Viability Reagent

The proliferation of hMSCs in fibrin gels (25,000 cells/gel) over time was quantified by adding 200 μL PrestoBlue^®^ cell viability reagent to each gel; cells grown on glass coverslips (25,000 cells/coverslip) served as a control. Viability measurements were taken following manufacturer’s instructions, briefly, media was replaced with a 10% PrestoBlue^®^ in media solution and incubated for 2 h at 37 °C/5% CO_2_. Following incubation, readings were taken at 560 nm excitation and 590 nm emission using a Perkin Elmer LS 50 B luminescence spectrometer (Perkin Elmer, Seer Green, UK) at day 1, 3, and 7. The PrestoBlue^®^ solution was then aspirated and fibrin gels were washed in PBS twice for 10 min before replacement with media and incubated at 37 °C/5% CO_2_ until the next required reading. Readings taken were standardised against the media control before estimation of cell number using calibration curves obtained using manufacturer’s instructions.

### 2.7. Spheroid Outgrowth Studies

The migration potential of hMSC spheroids (50,000 cells) within fibrin gels was monitored with time-lapse microscopy. Briefly, a bilayer setup was used in which an initial 200 μL gel was deposited into a 48 well plate and allowed to gelate (Figure 1c). Spheroids were generated following an adapted micro-mass method from Ahrens et al. [50] in agarose [51]. 1.5% agarose gels were prepared and autoclaved prior to pipetting 50 µL/well into flat bottom wells. After further sterilization under UV for 30 min a 50 µL cell suspension in hMSC media (as defined in Section 2.4) containing 50,000 cells was added to each well. Cells were then cultured at 37 °C/5% CO_2_ for 72 h to allow the spheroid to form. The spheroids were then embedded into 200 μL of fibrin gel which was deposited onto the initial base gel, followed by incubation at 37 °C/5% CO_2_ for a further 1 h. Spheroid outgrowth was monitored with a Nikon TiE inverted microscope equipped with a Nikon DS-Fi2 camera head (Nikon Instruments, Kingston upon Thames, UK) and a cage incubation system (Okolab, Pozzuoli, Italy) to provide environmental control at 37 °C/5% CO_2_. Images were acquired with Nikon NIS Elements software (Nikon Instruments) at 40× magnification in 1 h intervals for 72 h and analysed using ImageJ 1.50i (National Institute of Health, Bethseda, Maryland, USA) equipped with the LOCI Bio-Format plugin. A migration index was calculated for each individual spheroid examined as follows:

(1) $$\;Migration\;index=\;\frac{Total\;area-spheroid\;core\;area}{Total\;area}\;$$

### 2.8. Statistical Analysis

Quantitative data were statistically analysed using GraphPad Prism (GraphPad, Version 6.01, La Jolla, CA, USA). Data were assessed for normality prior to performing one-way (repeated measures where required) or two-way analysis of variance (ANOVA) with Tukey’s and Sidak’s test for comparison. Significance was set at *p* ≤ 0.05 (*), *p* ≤ 0.01 (**) and *p* ≤ 0.001 (***).

## 3. Results

### 3.1. SEM Analysis

**Figure 2:**
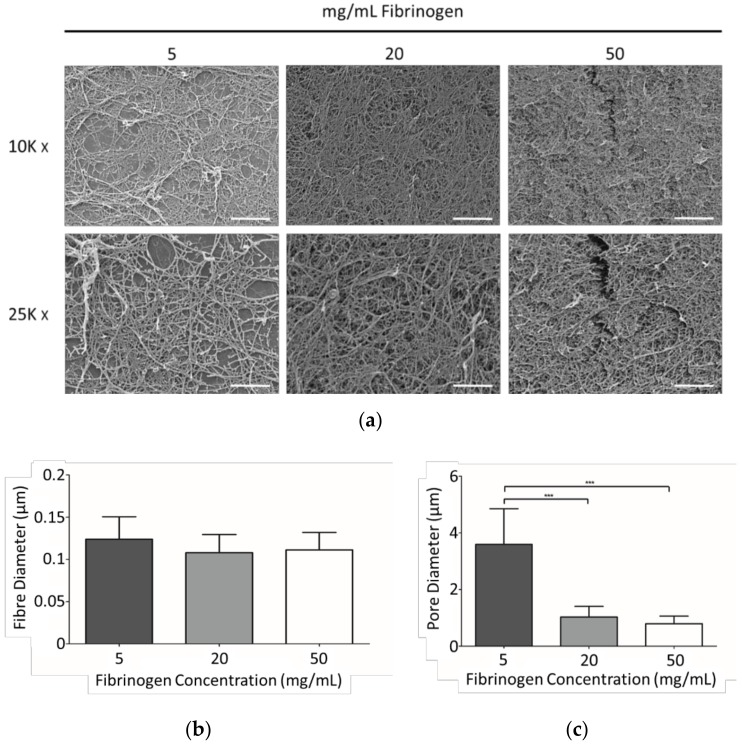
(**a**) Representative SEM micrographs of fibrin gels comprising 5, 20, and 50 mg/mL fibrinogen, and 5 U/mL thrombin at 10 K and 25 K magnification. Scale bars at 10 K magnification represent 5 μm; scale bars at 25 K magnification represent 2 μm. (**b**) Fibre diameter and (**c**) pore diameter distributions estimated from image analysis on two representative images. *p* ≤ 0.001 (***).

SEM micrographs were obtained of fibrin gels comprising 5, 20, and 50 mg/mL fibrinogen, and were analysed to assess fibre and pore sizes (Figure 2). The higher fibrinogen concentrations resulted in increased fibre density with smaller average pore sizes when compared to the more open structure depicted at the lowest fibrinogen concentration. The diameter of the nanofibres did not vary with fibrinogen concentration.

### 3.2. Viscoelasticity of Fibrin Gels

**Figure 3:**
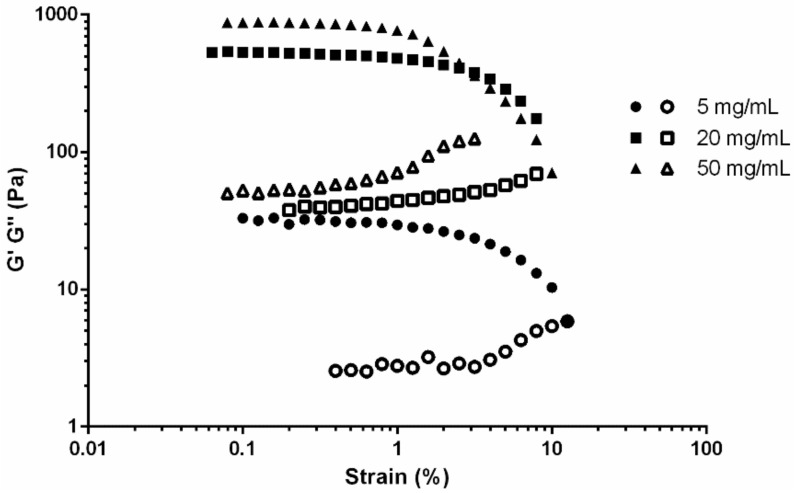
Representative strain sweeps of fibrin gels comprising 5 (circles), 20 (square), and 50 mg/mL (triangles) fibrinogen and 5 U/mL thrombin. Filled symbols indicate storage modulus (G’) and hollow symbols indicate loss modulus (G’’).

The rheological results were consistent, with variations between replicates of less than 5%. Across the range of fibrinogen concentrations, the storage/elastic modulus (G’) was greater than the loss/viscous modulus (G’’) until gel disintegration, indicating the elastic behaviour of fibrin gels (Figure 3). The G’ of the fibrin gels increased non-linearly with fibrinogen concentration, with a more marked increase from 5 mg/mL to 20 mg/mL than from 20 mg/mL to 50 mg/mL. In general, moduli appeared stable up until around 1% strain, which determined the linear viscoelastic region prior to this value.

### 3.3. Viability and Morphology of hMSCs within Fibrin Gels

**Figure 4:**
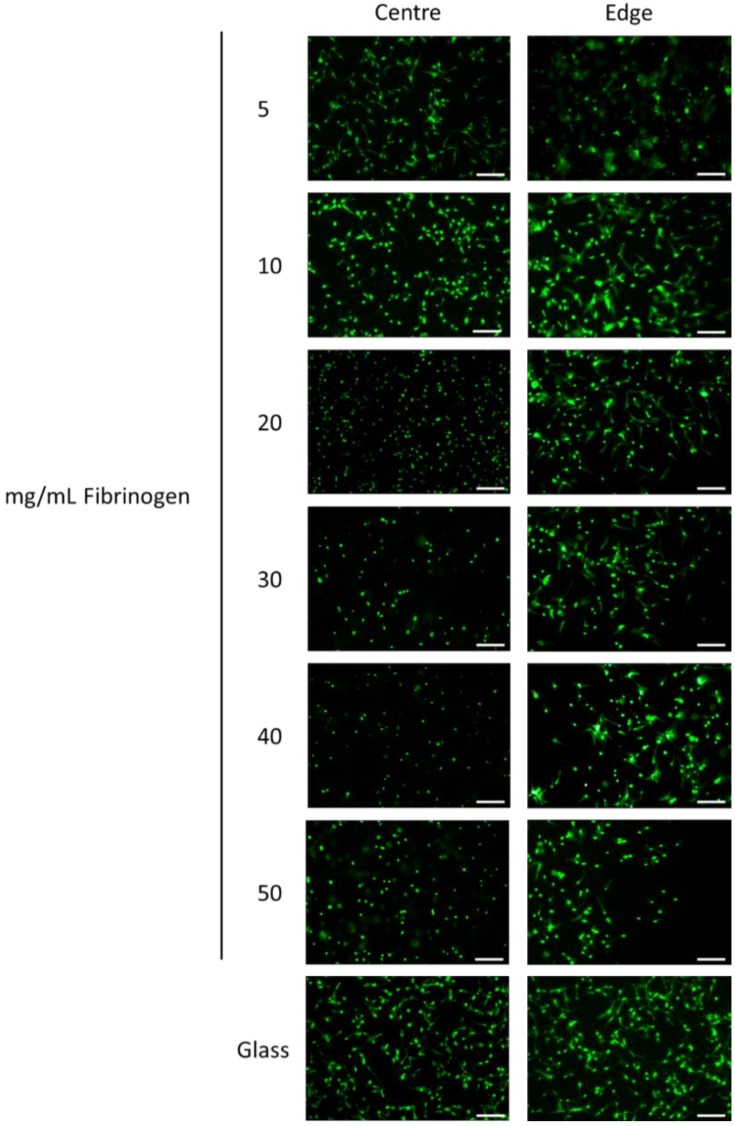
Representative images, at the center and edge, of cell-laden fibrin gels stained with calcein-AM (green) and ethidium homodimer-1 (red) (live/dead). Fibrin gels comprised 5, 10, 20, 30, 40, and 50 mg/mL fibrinogen and 5 U/mL thrombin. Cells seeded onto borosilicate glass coverslips served as controls. Scale bar equates to 200 μm.

The viability and morphology of hMSCs within fibrin gels at initial encapsulation was assessed after 6 h (Figure 4). For each concentration, cells displayed low cytotoxicity, good viability and an even distribution within the gels. Cells within the centre of fibrin gels at lower fibrinogen concentrations (5 mg/mL and 10 mg/mL) displayed an elongated, spindly morphology, similar to the glass control, as opposed to the smaller, rounder appearance found at higher concentrations. At the edges of the gels, cells also exhibited an elongated morphology at higher fibrinogen concentrations.

### 3.4 Encapsulated hMSC Proliferation

**Figure 5:**
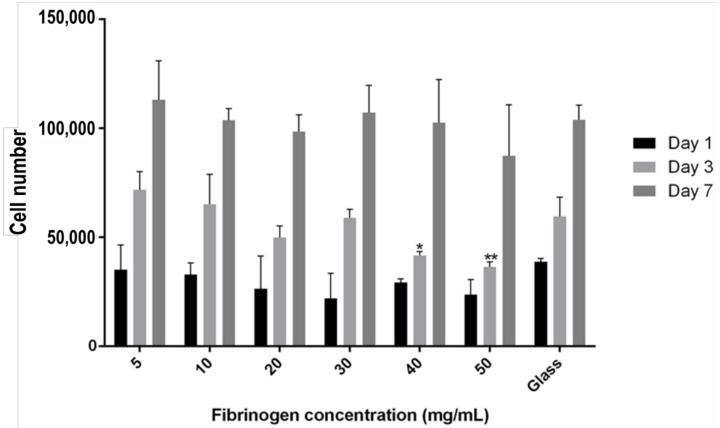
Proliferation of hMSCs within fibrin gels at day 1 (black), 3 (light grey), and 7 (dark grey) as quantified by PrestoBlue^®^ cell viability assay. Fibrin gels comprised 5, 10, 20, 30, 40, and 50 mg/mL fibrinogen and 5 U/mL thrombin. Data is expressed as mean ± SD (*n* = 3). * *p* < 0.05, ** *p* < 0.01 to the glass coverslip control.

The numbers of encapsulated hMSCs in fibrin gels was assessed over a 7-day period (Figure 5). No significant differences in cell number were observed at day 1 or day 7. At day 3, for all samples excluding those made with 40 mg/mL and 50 mg/mL fibrinogen, cell numbers had approximately doubled from day 1.

### 3.5 Encapsulated hMSC Migration

**Figure 6:**
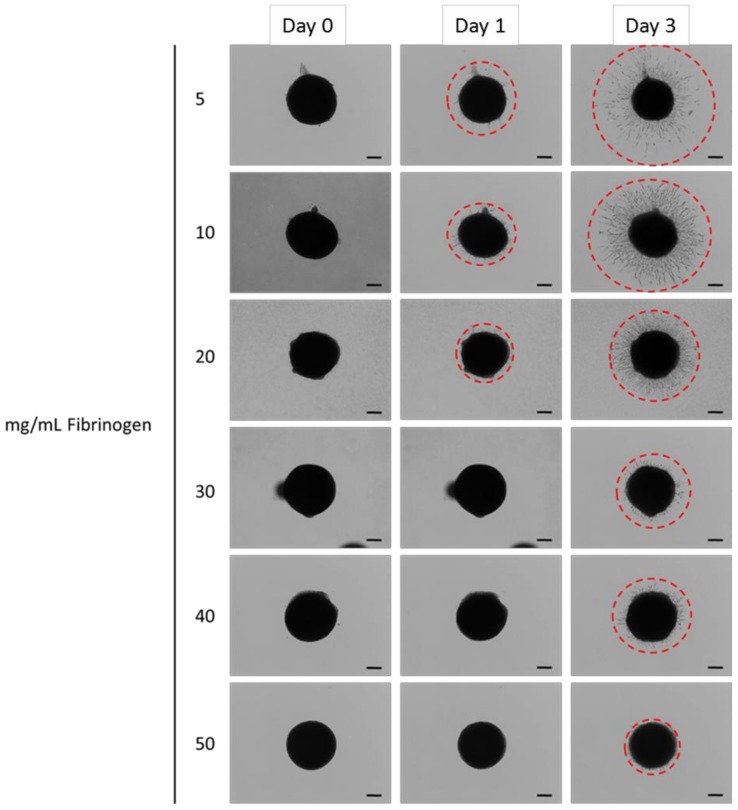
Representative images of spheroid outgrowth at day 0, 1, and 3. Gels comprised 5, 10, 20, 30, 40, and 50 mg/mL fibrinogen and 5 U/mL thrombin. Red dashed line approximates outgrowth at the greatest extent. Scale bar equates to 200 μm.

**Figure 7:**
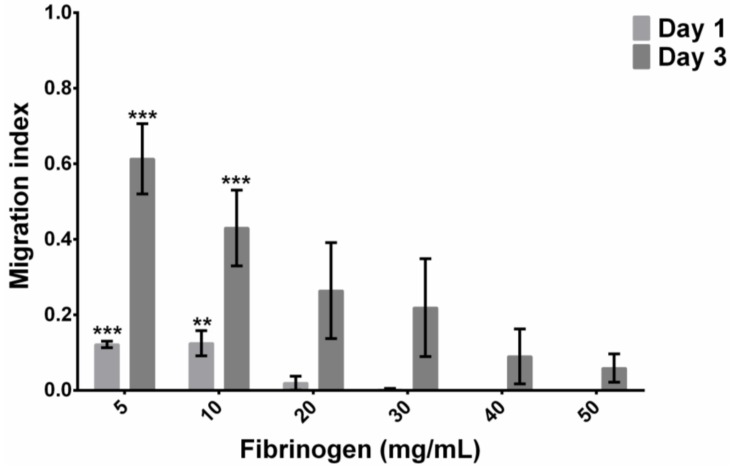
Migration index, at day 1 and 3, of hMSC spheroids within fibrin gels comprising 5, 10, 20, 30, 40 and 50 mg/mL fibrinogen and 5 U/mL thrombin. Migration index calculated as the total area subtracted by spheroid outgrowth area. Data represents mean ± SD (*n* = 3), ** *p* < 0.01, *** *p* < 0.001 to 50 mg/mL at respective time points.

Figure 6 shows representative images from the spheroid outgrowth experiements, and Figure 7 quantifies the results. Gels composed of 5 mg/mL to 20 mg/mL fibrinogen showed outgrowth at day 1, whereas spheroids in gels at 40 mg/mL and 50 mg/mL fibrinogen exhibited no outgrowth at this point. At day 3, gels comprising fibrinogen concentrations from 5 mg/mL to 30 mg/mL allowed a marked increase in hMSC migration compared to day 1. In particular, fibrin gels at 5 mg/mL presented a five-fold increase in outgrowth at day 3 which paralleled a notable reduction in spheroid core size. Spheroid outgrowth was observed at higher concentrations, although this was more limited. In all, results from the migration experiments demonstrated that increasing fibrinogen concentration in gels reduced the migratory capacity of encapsulated hMSCs.

## 4. Discussion

### 4.1 Fibrinogen Concentration Influenced Gel Structure

The 5% fibrinogen concentration resulted in a significantly increased average pore size when compared to the 20% and 50% concentrations (Figure 2). Higher fibrinogen concentrations provide more substrate for thrombin and thus increase fibre density, thought to be due to the presence of more fibres as opposed to an increase in the occurrence of branching [52]. The estimated fibre diameters are in line with previous fibre quantification studies for these concentrations of fibrinogen [52], thrombin [35,53], and Ca^2+^ [54], and overall the observation would be that, within the scope of this experimental study, varying the fibrinogen concentration primarily affects pore size, but that this is not a linear relationship.

### 4.2 Rheological Characterisation of Fibrin Gels

Higher concentrations of fibrinogen produced more rigid gels (Figure 3), with the relationship between G’ and fibrin concentration approximately linear. This concentration-dependent stiffness has been corroborated at lower [52] and higher [14] fibrinogen concentrations, and is thought to be caused by the increase in fibre density and general protein mass rather than a result of changes in individual fibre structure [52].

### 4.3 Cytocompatibility of Fibrin Gels

Live/dead staining demonstrated high cytocompatibility during initial cell dispersion (Figure 4). While viability was unchanged at different fibrinogen concentrations, divergent cell morphologies appeared between gels at low and high fibrinogen concentrations—a distinction that has been previously demonstrated with bone marrow stromal cells [16,40], and lipoaspirate-derived hMSCs [55]. Overall cells exhibited greater spread at the edges of gels, and in the centre of gels for the lower concentrations (notably for 5 mg/mL fibrinogen, but to a lesser extent for 10 mg/mL fibrinogen). The concentration dependent element of this is considered simply to be that the lower concentrations of fibrinogen allow cells to be more mobile and to spread out more easily. The reason for the edge effect is less clear, but at the edges of the gels there is a greater surface area in contact with media relative to the volume of gel. This means that:

(a) Cells at the edge could be able to obtain more nutrients and oxygen via diffusion and mass transport as a result [27], thus making them more mobile.
(b) Greater diffusion of media into the gels at the edge may make the gels locally less stiff, and allow greater spreading, possibly accentuated through cells accessing plasmin, allowing for some matrix remodelling.

Whilst of note the effect was not significant in terms of cytocompatibility, as at the centre of the gels all the gel concentrations showed excellent cytocompatibility.

### 4.4 Spheroid Outgrowth Experiments

Increasing fibrinogen concentration clearly diminished cell migration (Figure 7). The reduced migration at higher fibrinogen concentrations is considered to be primarily related to the confined porosity observed in these gels. Transmigration investigations within collagen have indicated the prominence of porosity in allowing fibrosarcoma cell migration [56], with peak migration occurring when pore size correlated with the cell body, with restrained migration contingent on pores that prevent nuclei from traversing through fibre networks [56,57]. Although these studies were performed within collagen gels, similarities in gel structure are observed with fibrin as both are fibrous protein-based 3D ECMs. The nuclei of the hMSCs used in this study are of the order of 10 µm diameter, and consideration of Figure 2 would then suggest that the cells can more easily make progress through a loose network with pores of around 2–5 µm across (5 mg/mL fibrinogen), than a tighter network with pores which are predominantly below 1 µm across (20 and 50 mg/mL fibrinogen). The center images in Figure 3 show that at the 5 mg/mL fibrinogen gel allowed cell spreading throughout the gel, which means that the cell migration process of cytoskeletal projection, followed by attachment onto the matrix, followed by posterior detachment [24,29] is much easier at that concentration. With easier cytoskeletal projection, and more space to move in, we assume that cells are able to “pull” their way through the fibre matrix at a much quicker rate.

The restricted migration at high fibrinogen concentrations has implications for tissue engineers who use commercial fibrin sealants for cell encapsulation. Products such as Tisseel^®^ are often used by surgeons, but their composition has a fibrinogen concentration in excess of 50 mg/mL. On the basis of the data presented here, these materials will not support the migration of encapsulated cells through the bulk material, with concentrations of 5 mg/mL or 10 mg/mL fibrinogen much better able to support migration. Sealants produced with higher fibrinogen concentrations can be used to immobilise cells or delay migration. Interestingly Hale’s et al. [46] 2D migration study with fibrin gels indicated that a fibrinogen concentration of 50 mg/mL was a threshold below which high magnitude migration could occur. This suggests that migration through a porous 3D network is significantly underestimated by 2D cell outgrowth models, and means that models of the type presented here offer a useful tool in determining hydrogel formulation for cell carrier applications.

### 4.5 Limitations of the Current Study

The characterisation of outgrowth within this paper has been through assessing the extent of maximum outgrowth, as interpreted from a 2D image of a 3D outgrowth model, so whilst the model is 3D the interpretation remains 2D. Other authors have characterized the outgrowth area [45], again from a 2D image of a 3D outgrowth model, and image analysis tools could provide a wide range of potential measures of outgrowth, including, for example, cell migration speed. Figure 6 shows that for this study the extent of maximum outgrowth seems representative of overall outgrowth, but image analysis could aggregate individual cell behaviour to produce a more detailed measurements than the technique used here, which could potentially be biased by the most active cell. The various parameters which could be used to interpret outgrowth images all offer different views of the same underlying process, and each will have advantages and disadvantages depending upon the intended application of the cell/gel construct. However, a key advantage of this type of model is that it gives a systematic, quantitative outcome which allows for lab to lab comparisons of cell behaviour in 3D environments. Further work with the outgrowth model will extend the technique to assess further materials for cell carrier applications, in particular for bio-ink formulation for 3D bioprinting, and will assess the value of different parameters for characterizing outgrowth.

## 5. Conclusions

This study has used a spheroid outgrowth model to provide clear and quantified information on the mobility of cells within fibrin gels, and we consider that the spheroid outgrowth model offers an excellent tool for the assessment of cell migration. The inverse relationship between fibrinogen concentrations and migration of encapsulated hMSCs within 3D fibrin gels has been previously reported, but not previously quantified in a way which gives clear guidance on gel formulation. The relationship has implications for scaffold design strategies and in clinical settings with use of fibrin sealants. Cell migration requires both cytoskeletal projection and sufficient space for cells to move into, and decreasing fibrinogen concentration produced fibrin gels with larger pores and lower density and stiffness, thus enabling quicker cell migration.

## Open Data

Data supporting this publication is openly available under an ‘Open Data Commons Open Database License’. Additional metadata are available at: http://dx.doi.org/10.17634/122951-5.
